# Prognostic and immunological implications of cathepsin Z overexpression in prostate cancer

**DOI:** 10.3389/fimmu.2025.1618487

**Published:** 2025-06-11

**Authors:** Junyue Tao, Yiding Chen, Xiaokang Bian, Tingting Cai, Changhao Song, Chaozhao Liang, Zongyao Hao, Jialing Meng, Qintao Ge, Jun Zhou

**Affiliations:** ^1^ Department of Urology, The First Affiliated Hospital of Anhui Medical University, Hefei, China; ^2^ Institute of Urology, Anhui Medical University, Hefei, China; ^3^ Anhui Province Key Laboratory of Urological and Andrological Diseases Research and Medical Transformation, Anhui Medical University, Hefei, China; ^4^ Department of Urology, Fudan University Shanghai Cancer Center, Shanghai, China; ^5^ Department of Oncology, Shanghai Medical College, Fudan University, Shanghai, China; ^6^ Shanghai Genitourinary Cancer Institute, Fudan University, Shanghai, China

**Keywords:** cathepsin Z, prostate cancer, expression profile, prognosis, immune infiltration, immune checkpoint, tumor mutation burden

## Abstract

**Background:**

Recent studies have underscored the potential involvement of cathepsin Z (CTSZ) in modulating the progression of diverse tumor types. Nevertheless, its specific role in prostate cancer (PCa) remains insufficiently understood. This study aimed to investigate the expression profile of CTSZ in PCa and evaluate its prognostic significance.

**Methods:**

Three independent cohorts, including TCGA-PRAD, MSKCC, and a real-world AHMU-PC cohort were enrolled in this study. Multidimensional strategies consist of spatial transcriptome analysis, differential expression analysis, survival analysis, and correlation with clinicopathological features were performed. Immunohistochemical staining and multiplex immunofluorescence staining were performed to evaluate the expression and spatial distribution of CTSZ and immune-related markers in PCa tissues. Functional studies were conducted through a series of experiments, including CCK-8 assay, colony formation, wound healing, and Transwell migration assays. ssGSEA and CIBERSORT algorithms immune infiltration evaluation, and GISTIC2.0 and MutSigCV for tumor mutation burden. Gene Set Enrichment Analysis was performed to identify potential signaling pathways involved.

**Results:**

CTSZ is highly expressed in PCa tissues and is associated with higher Gleason scores, advanced T/N staging, and poor prognosis. Survival analyses across multiple cohorts indicate that high CTSZ expression predicts shorter progression-free survival and overall survival. *In vitro* experiments showed that CTSZ knockdown suppresses PCa cell proliferation, invasion, migration, and colony formation. Immune profiling revealed that high-CTSZ tumors exhibit an immune-enriched microenvironment, characterized by increased infiltration of regulatory T cells and M2 macrophages, suggesting an immunosuppressive state. Notably, despite this phenotype, PD-1 and PD-L1 levels were also elevated in high-CTSZ tumors, indicating a potential role in immune checkpoint regulation. Additionally, high CTSZ expression was correlated with increased tumor mutation burden, particularly enriched for *TP53* and *SPOP* mutations. GSEA identified CAM, VEGF, and STAT signaling pathways as potential mechanisms through which CTSZ promotes tumor progression, highlighting its potential as both a prognostic biomarker and therapeutic target in PCa.

**Conclusions:**

Our study highlights CTSZ as a potential prognostic and therapeutic biomarker in PCa, demonstrating that its overexpression is associated with immune cell infiltration, immune checkpoint molecule expression (such as PD-1 and PD-L1), tumor mutation burden, and key oncogenic pathways. These findings suggest that CTSZ may serve not only as a predictor of patient prognosis but also as a promising indicator for immunotherapy response and personalized treatment strategies in PCa.

## 1 Introduction

In 2022, over 1,444,600 new cases were reported worldwide with prostate cancer (PCa), and PCa has become the dominant reason for cancer deaths for men (1). With the global population aging rapidly, the incidence of PCa is expected to continue rising in the coming decades, placing an increasing burden on public health systems and healthcare resources (2). Effective risk stratification and accurate prognostic assessment in PCa patients are crucial for guiding the appropriate treatment strategies for PCa (3). Currently, clinicians primarily rely on clinical parameters, such as prostate-specific antigen (PSA) levels, imaging assessments, and histopathological evaluations (including the Gleason score), to guide treatment decisions for patients with PCa (4, 5). Unfortunately, these clinical parameters fail to provide a definitive prognosis for individual patients, often resulting in two extreme outcomes: under-treatment and over-treatment (6). Therefore, exploring the reliable biomarkers of PCa to guide the individual treatment has become the focus of PCa research.

Cathepsin plays a pivotal role in multiple tumorigenic processes, including angiogenesis, tumor growth, and invasion (7). They reshape the tumor microenvironment by degrading the extracellular matrix and activating various growth factors, cytokines, and chemokines (8, 9). Notably, cathepsins facilitate tumor invasion and metastasis by disrupting intercellular adhesion molecules (10). Among them, Cathepsin Z (CTSZ), also known as Cathepsin X or P, is distinguished by its dual mono- and dipeptidyl carboxypeptidase activities (11). CTSZ primarily cleaves and activates molecules such as lymphocyte function-associated antigen (LFA) and Steroid Receptor Coactivator (SRC) kinase. LFA cleavage alters cellular morphology and enhances motility, linking CTSZ to cancer progression and metastasis (11). Additionally, CTSZ promotes T cell adhesion and migration across the endothelium within the tumor microenvironment, thereby influencing CD8^+^ T cell infiltration—a well-recognized marker of favorable prognosis and prolonged survival across multiple cancers (12). The CTSZ gene is located on chromosome 20q13, a region frequently amplified in various malignancies (13). Previous studies have associated CTSZ overexpression with several solid tumors, including gastric (14), lung (15), liver (16), and breast cancer (17). Recent evidence further suggests that CTSZ may also play an important role in PCa. For instance, proteomic analyses have identified CTSZ as a stratifying biomarker across different PCa Gleason grades (18), and its expression in peripheral blood has been proposed as a complementary biomarker to PSA for improving diagnostic accuracy and reducing unnecessary prostate biopsies (19). Furthermore, Mendelian randomization analyses have indicated a genetic association between CTSZ and cancer risk, including PCa (20).

Despite growing evidence of CTSZ involvement in various cancers, its expression pattern and clinical significance in PCa remain poorly defined. In this study, we integrated data from multiple public and real-world PCa cohorts and applied bioinformatics analyses, immunohistochemistry, and *in vitro* functional assays to systematically evaluate the prognostic, immunological, and biological relevance of CTSZ. Our findings suggest that CTSZ may serve as a promising biomarker for personalized prognosis assessment and therapeutic stratification in PCa.

## 2 Materials and methods

### 2.1 Raw data collection

This study incorporated three independent PCa cohorts. The Cancer Genome Atlas Prostate Adenocarcinoma (TCGA-PRAD) cohort, sourced from the Genomic Data Commons (GDC) platform (https://portal.gdc.cancer.gov/), comprised 496 patients with both gene expression profiles and clinical data. Additionally, the Memorial Sloan Kettering Cancer Center (MSKCC) Prostate Adenocarcinoma cohort, comprising 140 samples, was retrieved from the MSKCC database (http://www.mskcc.org/mskcc/). Furthermore, a real-world PCa cohort, designated as the Anhui Medical University Patients Cohort (AHMU-PC) (21), was derived from our previous research and approved by the ethics committee of the First Affiliated Hospital of Anhui Medical University. This cohort includes gene expression and clinical data from 69 patients who underwent prostatectomy at our hospital between 2019 and 2021, detailed clinical characteristics are provided in **Supplementary Table S1**. For analytical purposes, the TCGA-PRAD cohort was utilized as the training set, while the MSKCC and AHMU-PC cohorts served as validation sets. All gene expression data were annotated using the corresponding platform, underwent log2(x + 1) transformation, and were expressed as fragments per kilobase per million reads (FPKM). Patients were stratified into high and low expression groups based on the median CTSZ expression level. The clinicopathological characteristics of the three cohorts are detailed in **Supplementary Table S2**. In addition, CTSZ expression across multiple cancer types, including PCa, was examined using TCGA data from the UCSC Xena platform (https://xenabrowser.net/datapages/) (22).

### 2.2 Analysis of the influence of CTSZ expression on the prognosis of PCa

To investigate the association between CTSZ mRNA expression and clinicopathological features in PCa, we analyzed clinical and transcriptomic data from 496 patients in the TCGA-PRAD cohort. This analysis was validated in two independent cohorts: 140 patients from the MSKCC dataset and 69 patients from the AHMU-PC cohort. Patients were stratified into clinical subgroups for comparison. Differences in CTSZ expression among these subgroups were assessed using the ‘limma’ R package (23). Survival analysis based on CTSZ expression was performed using the GEPIA2 platform (http://gepia2.cancer-pku.cn/) (24).

### 2.3 Immunohistochemistry and multiplex immunofluorescence staining for CTSZ and immune markers

To assess the expression level of CTSZ in PCa tissues and its spatial relationship with key immune components within the tumor microenvironment, both immunohistochemistry (IHC) and multiplex immunofluorescence (mIF) staining were performed using the same tissue microarray (HPro-Ade045PG-01, OUTDO IVD, Shanghai), which includes 41 PCa tumor specimens and 3 matched normal prostate tissues. IHC staining was carried out using anti-CTSZ antibody (Cat. ab182575, Abcam, UK) and anti-PD-1 antibody (Cat. ab52587, Abcam, UK), following a protocol adapted from our previously published method (25). Semi-quantitative scoring was used to evaluate expression levels, based on both the percentage of positively stained cells (0: negative; 1: 1–10%; 2: 11–50%; 3: 51–80%; 4: >80%) and staining intensity (0: no staining; 1: weak; 2: moderate; 3: strong). mIF staining was conducted to further explore the spatial relationships between CTSZ and immune checkpoint molecules (PD-1, PD-L1), as well as CD163^+^CD206^+^ M2 macrophages. The following primary antibodies were used: anti-CTSZ antibody (Cat. ab182575, Abcam, UK), anti-PD-1 antibody (Cat. ab52587, Abcam, UK), anti-PD-L1 antibody (Cat. ab213524, Abcam, UK), anti-CD163 antibody (Cat. ab182422, Abcam, UK), and anti-CD206 antibody (Cat. ab64693, Abcam, UK). Staining was performed using the PANO 7-plex multiplex immunohistochemistry kit (Panovue, China), following the manufacturer’s instructions. Imaging was conducted using the Mantra Quantitative Pathology Imaging System (Akoya Biosciences), and multispectral images were analyzed with inForm software (v2.4.8, Akoya Biosciences).

### 2.4 Cell culture and transfection

Human prostate cell lines RWPE-1, PC-3, DU145, and LNCaP were obtained from the Shanghai Cell Bank (Shanghai, China). RWPE-1 cells were cultured in Keratinocyte Serum-Free Medium (K-SFM), while PC-3, DU145, and LNCaP cells were maintained in RPMI 1640 medium supplemented with 10% fetal bovine serum (FBS), 1% L-glutamine, and 1% penicillin-streptomycin (all from Gibco^®^, Shanghai, China). All cells were maintained at 37°C in a humidified incubator with 5% CO2. To knockdown CTSZ expression, three siRNAs were designed (sequences listed in **Supplementary Table S3**). PC-3 and DU145 cells were seeded into six-well plates (3 × 10^5^ cells per well). When cell confluence reached approximately 70%, transfection was performed using Lipofectamine™ 3000 Reagent (Thermo Fisher Scientific, USA) according to the manufacturer’s protocol. After 48 hours, cells were harvested for subsequent functional assays.

### 2.5 Western blot and RT-qPCR

After cell collection, mRNA and protein expression levels assessed by using RT-qPCR and Western blotting (WB), respectively, following our previously published protocols (26–28). The antibodies used included rabbit anti-CTSZ monoclonal antibody (Cat. 16578-1-AP, Proteintech, USA), and rabbit anti-Tubulin polyclonal antibody (Cat. 10068-1-AP, Proteintech, USA). Primer sequences for RT-qPCR are listed in **Supplementary Table S3**.

### 2.6 Cell proliferation, invasion, and migration assays

The functional roles of CTSZ in cell proliferation, invasion, and migration were assessed using CCK-8, colony formation, Transwell, and wound healing assays, respectively, as described in our previous studies (26–28).

### 2.7 Enrichment analyses

To investigate the potential molecular mechanisms and relevant pathways associated with CTSZ expression in PCa, patients from the TCGA-PRAD cohort were stratified into high and low CTSZ expression groups based on the median expression level. Gene Set Enrichment Analysis (GSEA) was conducted to identify CTSZ-related gene sets, with 1000 permutations performed to calculate normalized enrichment scores and enrichment significance. Enrichment analyses for Gene Ontology (GO), Kyoto Encyclopedia of Genes and Genomes (KEGG), and HALLMARK pathways were conducted using the “org.Hs.eg.db” R package, with further functional annotation conducted through the “clusterProfiler” R package (29, 30). The results were visualized using the “enrichplot” R package (31). Statistical significance was defined by setting a P-value threshold of < 0.01 and a false discovery rate below 0.25. Additionally, in the analysis of immune status, we selected immune-related pathways from the c2.cp.kegg.v7.4 collection in the MSigDB database (https://www.gsea-msigdb.org/gsea/msigdb) (32), including Toll-like receptor signaling, γ-Fc-mediated phagocytosis, chemokine signaling, antigen processing and presentation, B cell receptor signaling, and T cell receptor signaling pathways. Furthermore, similar analyses were conducted for the P53 signaling pathway.

### 2.8 Tumor immune infiltration analysis

Single-sample Gene Set Enrichment Analysis (ssGSEA) was used to evaluate the immune cell activation status between CTSZ high- and low-expression groups in the TCGA-PRAD cohort. The ESTIMATE algorithm, available in the “estimate” R package, was applied to calculate immune and stromal scores (33), and Pearson correlation analysis was conducted to assess the relationship between CTSZ expression and these scores. The CIBERSORT algorithm was applied to assess immune cell infiltration and investigate the correlation between immune cell infiltration levels and CTSZ expression (34). Significant correlations were visualized using scatter plots. Between the high- and low-CTSZ expression groups, differences in the infiltration of B cells, macrophages, mast cells, plasma cells, and regulatory T cells were analyzed. Given the role of M2 macrophages in PCa, further validation was performed to examine the relationship between M2 macrophages and CTSZ expression.

### 2.9 Tumor mutation burden assessment

To gain deeper insights into the influence of CTSZ on gene mutations in PCa, we analyzed differences in focal and arm-level amplifications and deletions between high- and low-CTSZ expression groups. Gene copy number alteration data were retrieved from GISTIC2.0 via the GDAC Firehose platform (https://gdac.broadinstitute.org). To evaluate the tumor mutation burden, we determined the frequency of nonsynonymous mutations per million base pairs. Additionally, MutSigCV_v1.41 (www.broadinstitute.org) was applied using default settings to identify significant cancer-related mutations (q < 0.05) across the entire TCGA cohort. To identify significant mutation differences, we independently compared the high- and low-CTSZ expression groups. The “ComplexHeatmap” R package (35) was employed to generate an oncoprint, facilitating the visualization of mutation data.

### 2.10 Spatial tranciptome analysis

The spatial transcriptome (ST) data was sourced from the Genomics official website (https://www.10xgenomics.com/). The data were processed and analyzed using the Seurat R package (36). For data standardization, the SCTransform approach was implemented, which incorporated functions such as SelectIntegrationFeatures, PrepSCTIntegration, FindIntegrationAnchors, and IntegrateData to merge the ST datasets. The ‘Spatial’ analysis method, coupled with the ‘poisson’ model, was applied for standardizing sparse, count-based data. After standardization, the datasets were merged, and the DefaultAssay was set to ‘SCT’. Principal component analysis was conducted on the ‘SCT’ data to reduce dimensionality, followed by clustering using the Louvain algorithm with a resolution parameter of 0.6. To visualize the expression patterns of cells within the ST data, the SpatialDimPlot and SpatialFeaturePlot functions were employed. The Cottrazm (37) package was used to define the tumor boundary, which was then subdivided into three regions: malignant (Mal), boundary (Bdy), and non-malignant (nMal). SpatialPlot function was used to visualize the spatial distribution of CTSZ.

### 2.11 Statistical methods

All data analyses and statistical tests were executed using R (version 4.0.2). For continuous data, the Wilcoxon rank-sum test was employed when the distribution deviated from normality; otherwise, the student’s t-test was used for comparison. Categorical data were assessed using the Chi-square test, while Fisher’s exact test was employed when expected frequencies were low. Cox proportional hazards regression and the log-rank test were performed, with hazard ratios and their respective 95% confidence intervals estimated. Kaplan-Meier curves were used for visualization. A p-value under 0.05 was considered indicative of statistical significance.

## 3 Results

### 3.1 CTSZ expression is elevated in multiple cancers and associated with poor prognosis

**Figure 1:**
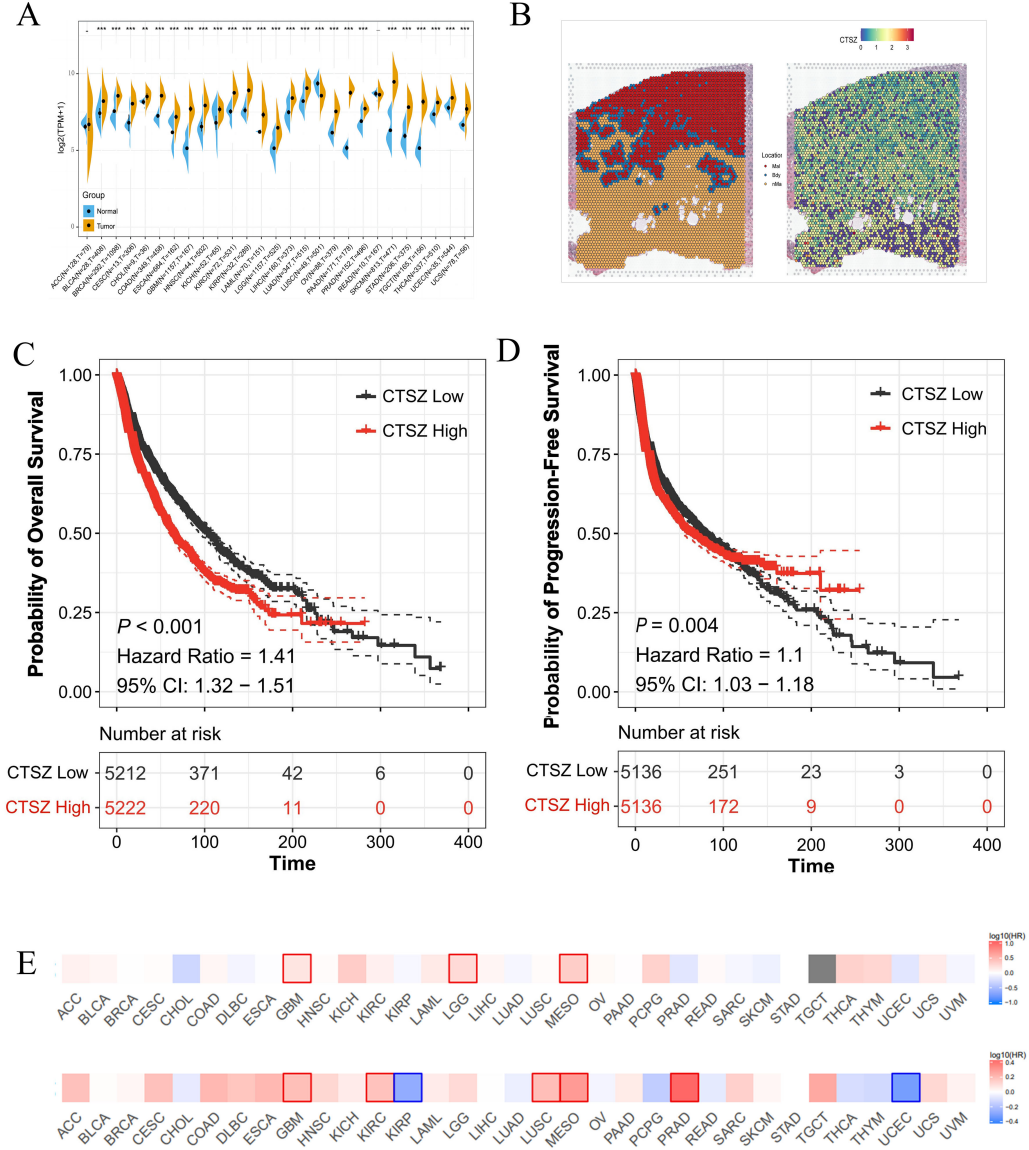
Expression and prognostic significance of CTSZ across multiple cancer types. **(A)** Differential expression analysis of CTSZ in 26 cancer types demonstrating significantly elevated expression in tumor tissues compared to normal tissues. **(B)** Spatial transcriptomic analysis reveals that CTSZ expression is markedly higher in Mal than in Bdy and nMal regions. **(C, D)** Kaplan-Meier analysis showing that high CTSZ expression correlates with poorer OS and shorter PFS. **(E)** High CTSZ expression consistently associates with reduced OS and PFS across multiple cancer types. * p < 0.05. ** p < 0.01. *** p < 0.001.

Pan-cancer analysis revealed significantly elevated CTSZ expression in 26 tumor types (all *P* < 0.05), with particularly high levels in PCa (**Figure 1A**). CTSZ expression was also higher in malignant regions compared to Bdy and nMal areas (**Figure 1B**). Patients were categorized into low- and high-CTSZ expression groups according to the median expression. Survival analysis indicated that patients with higher CTSZ expression experienced significantly shorter overall survival (OS) and progression-free survival (PFS) (*P* < 0.001) compared to those with lower expression levels. (**Figures 1C, D**). Pan-cancer analysis also demonstrated prognostic value in several malignancies, including glioblastoma, glioma, mesothelioma, renal carcinoma, lung squamous carcinoma, endometrial carcinoma, and PCa (all *P* < 0.05) (**Figure 1E**). Collectivaly, higher CTSZ implicated a poor clinical outcomes in the context of tumor.

### 3.2 High expression of CTSZ correlates with clinical staging and predicts poor prognosis in external cohorts

**Figure 2:**
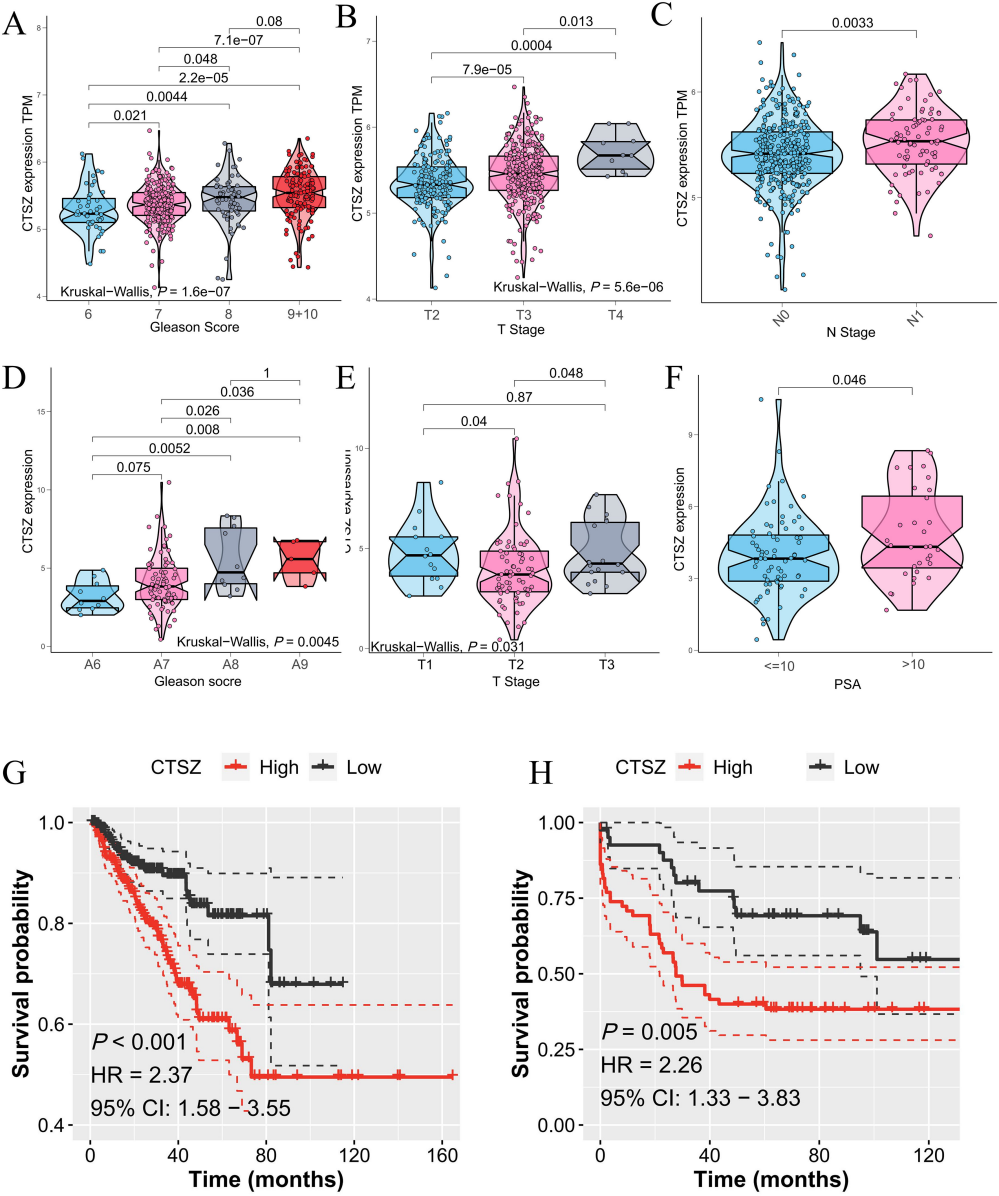
CTSZ expression and prognostic impact in PCa from TCGA-PRAD and MSKCC cohorts. **(A–C)** In the TCGA-PRAD cohort, CTSZ expression positively correlates with Gleason scores, T stage, and N stage. **(D–F)** In the MSKCC cohort, CTSZ expression positively associates with Gleason scores, T stage, and PSA levels. **(G, H)** Kaplan-Meier survival analysis indicates high CTSZ expression is linked with worse OS in both the TCGA-PRAD and MSKCC cohorts.

The association between CTSZ expression and clinicopathological parameters in PCa was analyzed using data from the TCGA-PRAD cohort. Higher CTSZ expression correlated with increased Gleason scores, T stages, and N stages (**Figures 2A-C**). For instance, patients with Gleason scores of 9 and 10 exhibited significantly higher CTSZ expression compared to those with a score of 6 (*P* = 2.2e-05). Additionally, CTSZ expression was significantly elevated in T4 stage patients relative to those in T2 stage (*P* = 0.0004). Similar findings were observed in the MSKCC cohort (**Figures 2D-F**), where high CTSZ expression was noted in patients with Gleason scores of 9 and 10 (*P* = 0.008) and PSA levels exceeding 10 ng/ml (*P* = 0.01). These results suggest that elevated CTSZ expression could be an indicator of poor prognosis in PCa. Furthermore, Kaplan-Meier analysis showed significantly shorter PFS in high CTSZ expression patients from both the TCGA-PRAD (*P* < 0.001) and MSKCC (*P* = 0.005) cohorts (**Figures 2G, H**), further supporting CTSZ as a potential risk factor in PCa.

### 3.3 High CTSZ levels assist in identifying tumor and gleason grade staging

**Figure 3:**
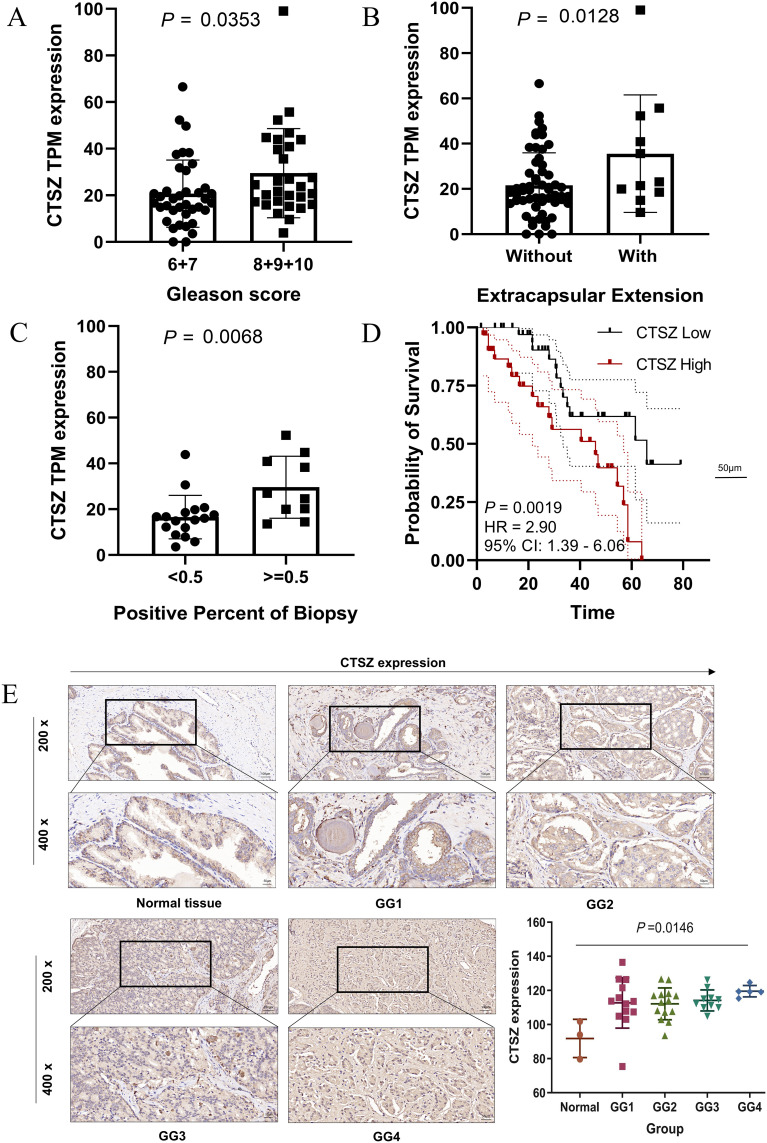
Validation of CTSZ expression and prognostic value in the AHMU-PC cohort and immunohistochemical analysis. **(A)** Higher CTSZ expression observed in PCa patients with Gleason scores of 8–10 compared to scores of 6–7. **(B)** Elevated CTSZ expression in PCa patients with extracapsular extension. **(C)** Increased CTSZ expression correlates with a biopsy-positive rate exceeding 50%. **(D)** Kaplan-Meier survival curve indicates significantly worse recurrence-free survival in patients with high CTSZ expression. **(E)** Representative immunohistochemical staining showing elevated CTSZ expression levels in PCa tissues.

We collected samples from 69 PCa patients along with their clinical data and conducted a long-term follow-up study (AHMU-PC) cohort. In this real-world cohort, patients with Gleason scores of 8, 9, and 10 exhibited significantly higher CTSZ expression compared to those with scores of 6 and 7 (*P* = 0.0353) (**Figure 3A**). Similarly, patients with extracapsular extension and those with a biopsy positivity rate exceeding 50% demonstrated higher CTSZ expression than their counterparts (*P* = 0.0218 and *P* = 0.0068, respectively) (**Figures 3B, C**). In survival analysis, patients with high CTSZ expression exhibited significantly shorter recurrence-free survival than those with low CTSZ expression (*P* = 0.0019) (**Figure 3D**). Interestingly, compared to normal tissues, CTSZ expression was significantly upregulated in PCa and increased notably with advancing malignancy (**Figure 3E**). These results indicated that CTSZ could be used as a novel biomarker for distinguishing tumors from normal prostate tissue and for risk stratification in PCa, highlighting its potential clinical value.

### 3.4 CTSZ knockdown inhibits proliferation, migration, and invasion in PCa cells

**Figure 4:**
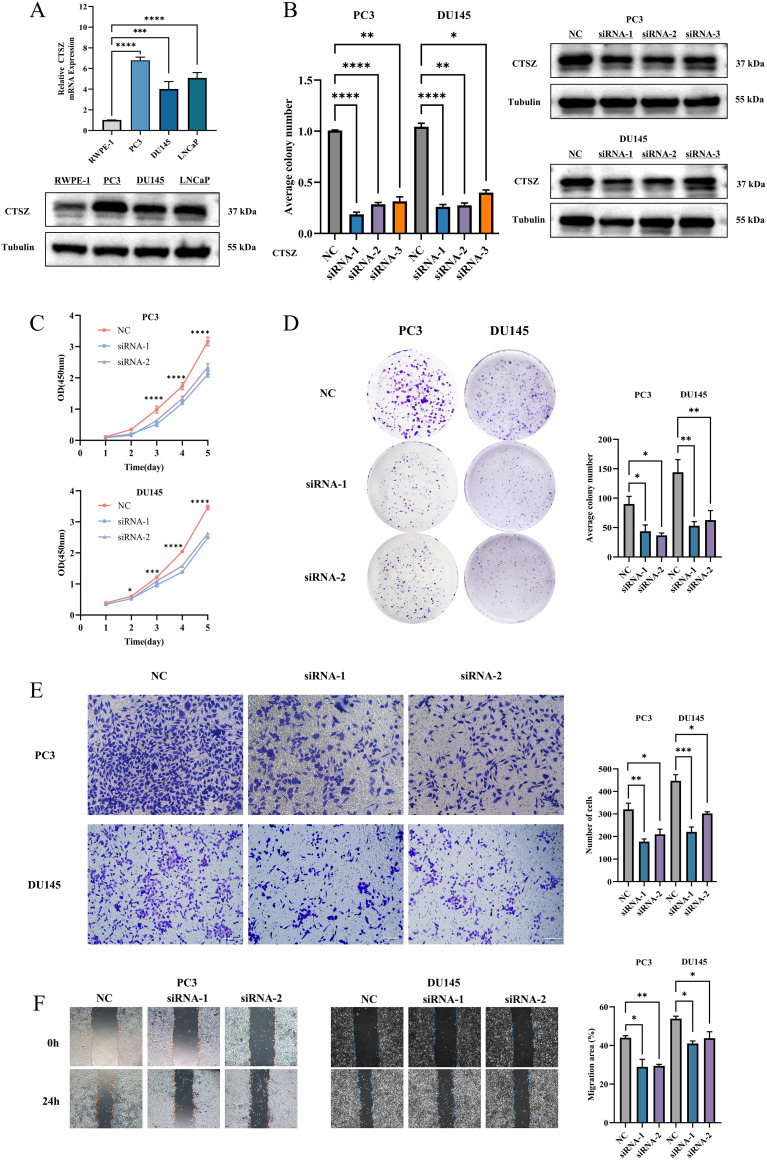
CTSZ expression in PCa cell lines and functional impact of CTSZ knockdown in PC3 and DU145 cells. **(A)** Comparative analysis of CTSZ expression in PCa cell lines versus normal prostate epithelial cells. **(B)** Validation of CTSZ knockdown efficiency in PC3 and DU145 cells by qPCR and WB analysis **(C–F)** CTSZ knockdown significantly suppressed cell proliferation **(C)**, colony formation **(D)**, invasion **(E)**, and migration **(F)** in both PC3 and DU145 cells. Data represent the mean ± standard deviation from three independent experiments (*P < 0.05, **P < 0.01, ***P < 0.001, ****P < 0.0001).

We assessed the mRNA and protein expression levels of CTSZ in RWPE-1 and three PCa cell lines (PC3, DU145, and LNCaP) using qPCR and Western blot analyses (**Figure 4A**). Among these cell lines, CTSZ expression was highest in PC3 cells, followed by DU145 and LNCaP. To assess the regulatory role of CTSZ in key biological processes of PCa cells, we silenced CTSZ expression in PC3 and DU145 cells using siRNA. Both qPCR and Western blot analyses confirmed the successful establishment of the cell model (**Figure 4B**). CCK-8 and colony formation assays demonstrated that CTSZ knockdown significantly suppressed the proliferative activity and colony formation ability of PC3 and DU145 cells (**Figures 4C, D**). In addition, Transwell assays revealed that CTSZ knockdown reduced the invasive capacity of PC3 and DU145 cells compared to the control group (**Figure 4E**). The wound-healing assay further showed that CTSZ knockdown impaired the migratory ability of PC3 and DU145 cells (**Figure 4F**).

### 3.5 High CTSZ expression correlates with immunosuppressive cell infiltration and immune checkpoint molecule expression

**Figure 5:**
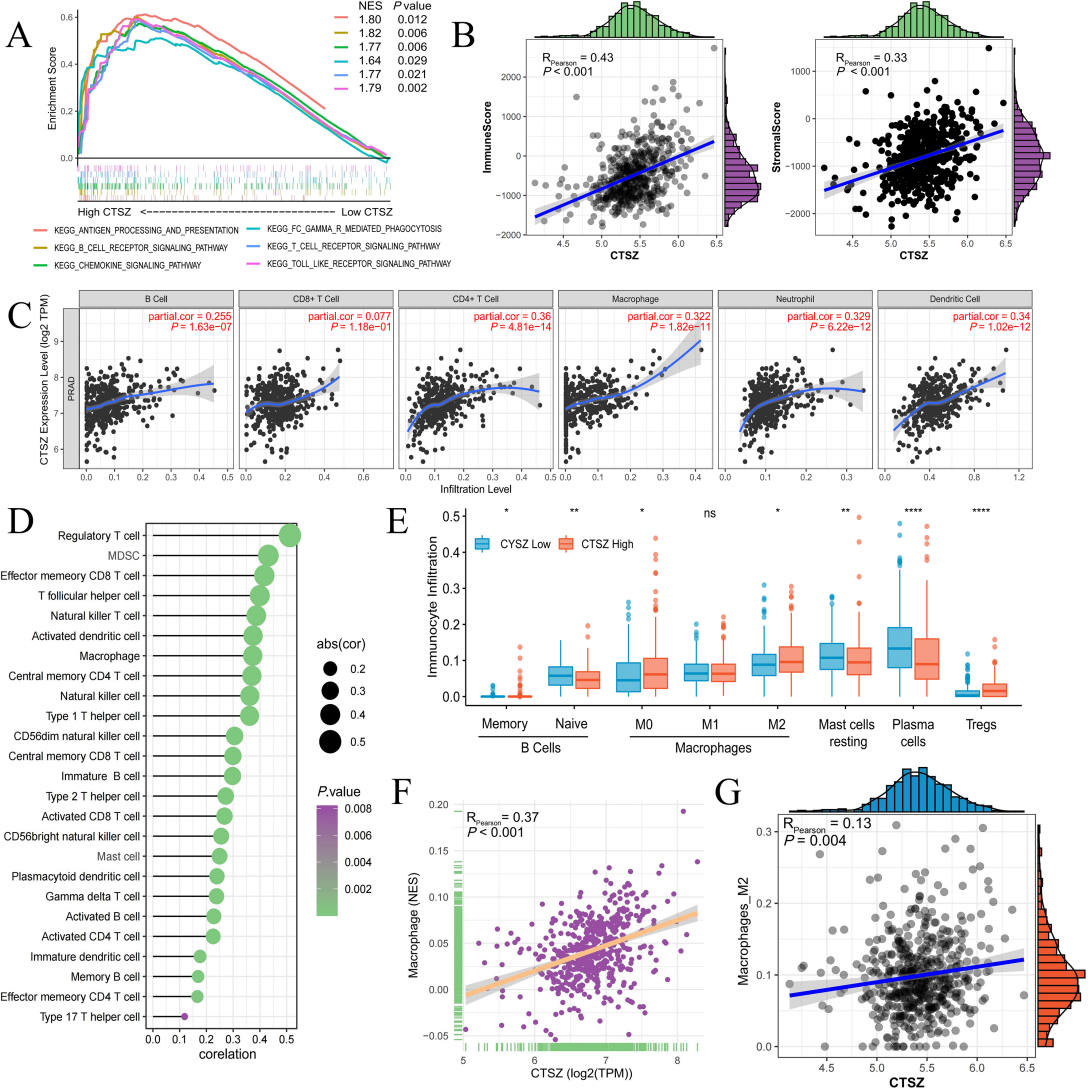
Relationship between CTSZ expression and tumor immune infiltration in PCa. **(A)** Pathway enrichment analysis revealing activation of immune-related pathways in high vs. low CTSZ expression groups. **(B)** CTSZ expression positively correlates with immuneScore and stromalScore. **(C, D)** Positive correlation between CTSZ expression and infiltration of multiple immune cell types in PCa. **(E)** Comparison of immune cell infiltration profiles between high and low CTSZ expression subgroups. **(F, G)** CIBERSORT analysis confirming positive correlation between CTSZ expression and infiltration levels of total macrophages and M2 macrophages. All data were obtained from the TCGA-PRAD cohort. ns: not significant (p ≥ 0.05). * p < 0.05. ** p < 0.01. **** p < 0.0001.

Using the ssGSEA algorithm, we found that high CTSZ expression in PCa patients correlates with the activation of several immune-related signaling pathways, including B cell receptor signaling, antigen processing, and T cell receptor signaling (**Figure 5A**). We also found that CTSZ expression was strongly correlated with ImmuneScore (*P* < 0.001) and StromalScore (*P* < 0.001), indicating a more abundant immune presence within the tumor microenvironment of PCa with high CTSZ expression (**Figure 5B**).

CTSZ expression showed a significant positive correlation with the infiltration of diverse immune cells, suggesting an overall immune-enriched tumor microenvironment. These immune cells included B cells, CD4^+^ T cells, neutrophils, macrophages, and dendritic cells (**Figures 5C, D**). Subgroup analysis further revealed a particularly strong association between CTSZ expression and the infiltration of regulatory T cells (Tregs) and M2-polarized macrophages, both of which are key mediators of immune suppression. This immune cell composition suggests that tumors with high CTSZ expression may possess an immunosuppressive microenvironment (**Figures 5E-G**).

**Figure 6:**
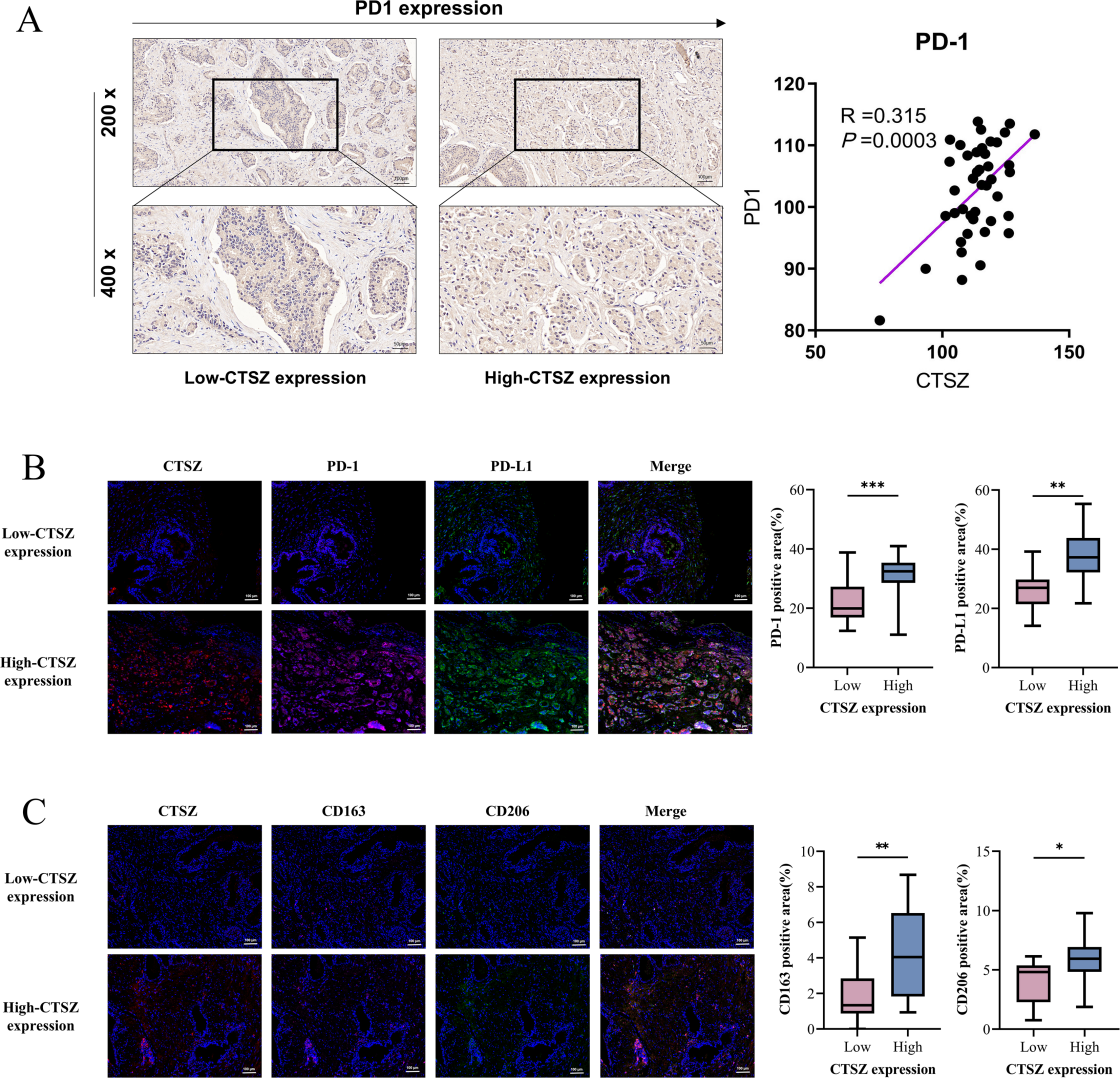
Spatial associations of CTSZ with immune checkpoint molecules and M2 macrophages in PCa tissues. **(A)** Immunohistochemistry results show a positive correlation between CTSZ and PD-1 expression in PCa tissues. **(B)** mIF images demonstrate spatial co-localization of CTSZ with immune checkpoint molecules PD-1 and PD-L1 in the tumor microenvironment. **(C)** mIF images reveal spatial proximity between CTSZ^+^ tumor cells and CD163^+^CD206^+^ M2 macrophages, suggesting a potential positive association. * p < 0.05. ** p < 0.01. *** p < 0.001.

Further analysis revealed that PD-1 expression was significantly elevated in the high CTSZ expression group, as determined by immunohistochemical staining, with a positive correlation observed between CTSZ and PD-1 expression levels (**Figure 6A**). mIF staining further confirmed the spatial co-localization of CTSZ with both PD-1 and PD-L1 in prostate cancer tissues (*P*< 0.05, **Figure 6B**), reinforcing their functional association within the tumor microenvironment. Moreover, adjacent to CTSZ^+^ tumor cells, more infiltration of CD163^+^CD206^+^ M2 macrophages were observed (*P*< 0. 05, **Figure 6C**), indicating a potential role for CTSZ in shaping an immunosuppressive niche. These findings suggest that, despite the presence of an immunosuppressive tumor microenvironment (TME), tumors with high CTSZ expression may remain susceptible to immune checkpoint blockade, particularly therapies targeting PD-L1 and PD-1.

### 3.6 High CTSZ expression is associated with high mutation load in PCa

**Figure 7:**
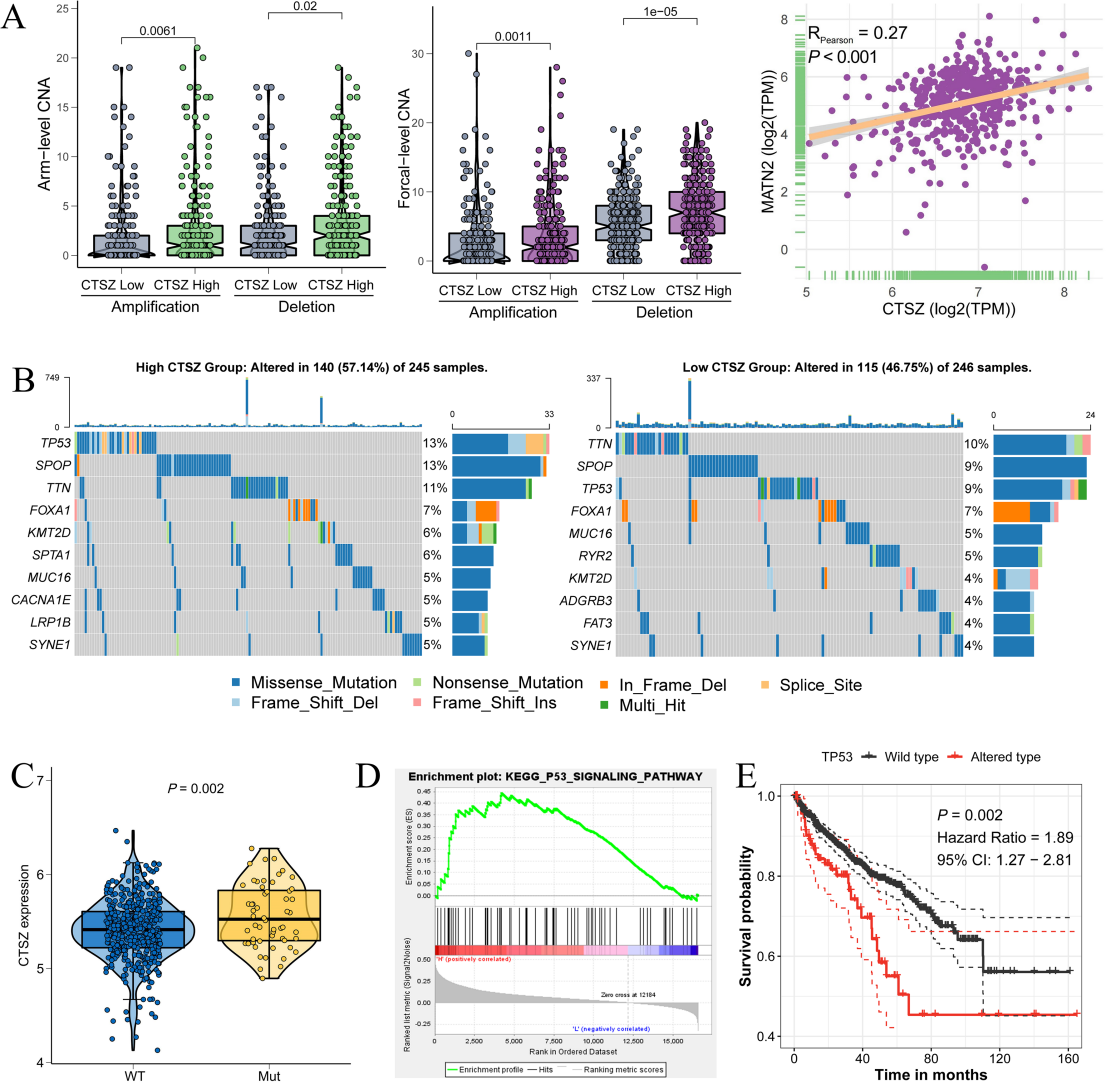
Association between CTSZ expression and genetic mutations in PCa. **(A)** Elevated arm-level and focal amplification/deletion events observed in the high CTSZ expression subgroup. **(B)** Comparative analysis of gene mutation frequencies between CTSZ high- and low-expression groups. **(C)** Significantly higher CTSZ expression in *TP53*-mutant PCa samples compared to *TP53* wild-type. **(D)** *TP53* signaling pathway enrichment identified in the CTSZ high-expression subgroup. **(E)** Kaplan-Meier analysis demonstrating worse prognosis in TP53-mutant PCa compared to wild-type cases. Genomic and transcriptomic data were retrieved from the TCGA-PRAD cohort.

Within the TCGA cohort, we conducted an analysis of mutation profiles in subgroups with high and low CTSZ expression. The high CTSZ expression group showed higher mutation burden, with increased amplifications and deletions (PArm-Amp = 0.0061, PFocal-Amp = 0.0011) (**Figure 7A**). MutSigCV analysis revealed a higher mutation frequency in the high CTSZ expression group (57.14%) compared to the low expression group (46.75%) (**Figure 7B**). Among the high CTSZ expression subgroup, *TP53* and *SPOP* had the highest mutation frequencies, whereas TTN was the most commonly mutated gene in the low CTSZ group.

In PCa, *TP53* mutations have been strongly linked to tumor progression, metastasis, and therapy resistance. PCa tumors with TP53 mutations showed significantly higher CTSZ expression than wild-type tumors (**Figure 7C**, P = 0.002). Furthermore, the *TP53* signaling pathway was enriched in the high CTSZ expression subgroup (**Figure 7D**). Overall, *TP53* mutations and signaling activation were strongly associated with high CTSZ expression. PCa patients with TP53 mutations showed poorer prognosis than those with wild-type *TP53* (**Figure 7E**, P = 0.002), consistent with our previous finding that high CTSZ expression predicts poor prognosis.

### 3.7 CTSZ promotes tumor progression via CAM, VEGF, and STAT signaling pathways

**Figure 8:**
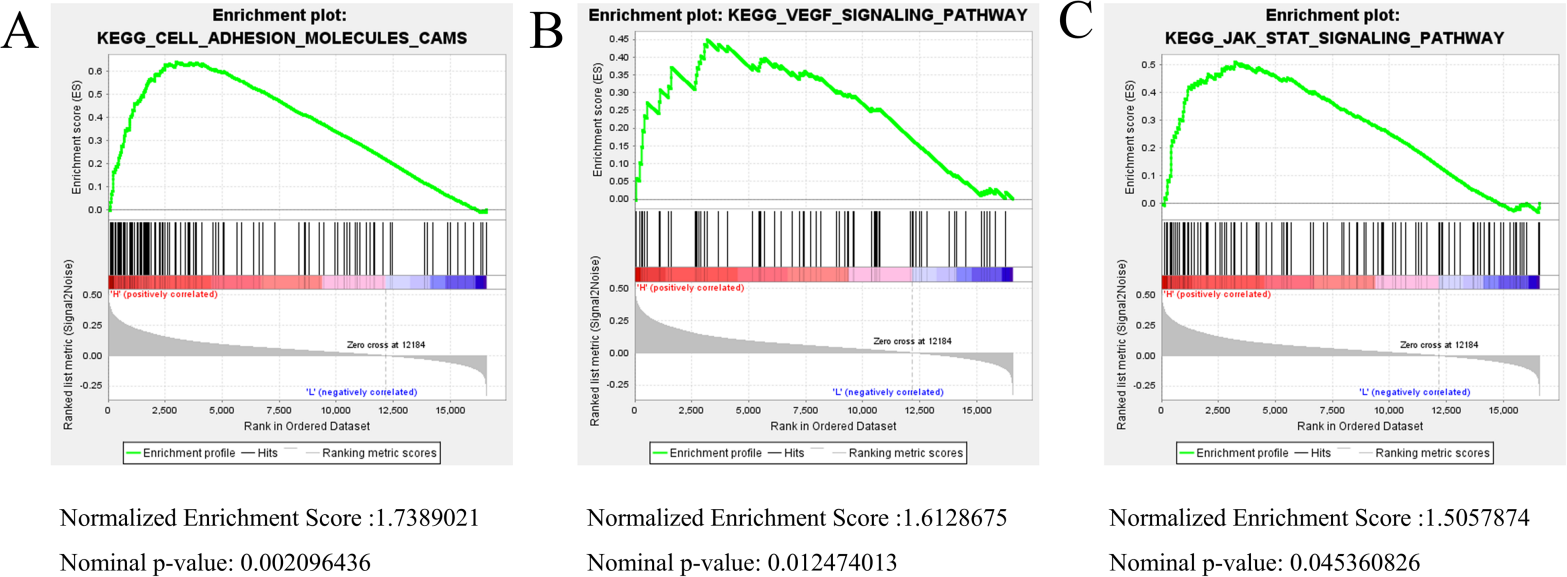
KEGG enrichment analysis linked to CTSZ expression. **(A–C)** KEGG enrichment analysis identified CAM, VEGF, and STAT signaling pathways as significantly enriched in association with high CTSZ expression. Enrichment analysis was based on gene expression data from the TCGA-PRAD cohort.

To investigate the potential pathways through which CTSZ may contribute to poor prognosis in PCa, we conducted KEGG enrichment analysis. The results identified several key pathways associated with CTSZ, including the cell adhesion molecule (CAM) signaling pathway (**Figure 8A**), the vascular endothelial growth factor (VEGF) signaling pathway (**Figure 8B**), and the signal transducer and activator of transcription (STAT) signaling pathway (**Figure 8C**). These pathways may underlie the poor prognosis associated with high CTSZ expression in PCa. They are well-known for promoting tumor progression, angiogenesis, and immune modulation, which may explain the unfavorable outcomes in PCa patients with elevated CTSZ expression.

## 4 Discussion

CTSZ is a lysosomal cysteine protease that has been implicated in tumor progression across various malignancies. While earlier studies have shown that CTSZ contributes to extracellular matrix degradation, cell adhesion and migration, and intracellular signaling (38, 39), its specific function and underlying mechanisms in PCa remain insufficiently characterized. In this study, we systematically profiled CTSZ expression in PCa by integrating bioinformatics analyses, spatial transcriptomics visualization, immunohistochemical staining, and *in vitro* functional assays. These approaches collectively advance our understanding of CTSZ’s oncogenic and immunomodulatory roles in the context of PCa.

Importantly, CTSZ demonstrates strong potential as a prognostic biomarker in PCa. Prior studies have shown that elevated CTSZ expression is significantly associated with unfavorable clinical outcomes in multiple malignancies, including renal cell carcinoma, colorectal cancer, and esophageal cancer (40–42). Consistently, our study reveals a significant correlation between high CTSZ expression and adverse clinicopathological characteristics, as well as poor prognosis, in PCa. Furthermore, functional experiments demonstrated that silencing CTSZ markedly inhibited the proliferation, migration, and invasion of PCa cells, aligning with its pro-tumorigenic roles reported in other cancer types. Collectively, these findings highlight CTSZ as a robust biomarker for prognostic assessment and risk stratification in PCa.

In recent years, immunotherapy has shown promising efficacy across various solid tumors, with its application in urological malignancies gradually expanding (43, 44). Given the notable heterogeneity of immune responses in PCa, the use of immune-related molecular biomarkers for more accurate patient stratification and personalized treatment has become a major focus of current research. The dynamic interaction between tumor cells and their surrounding microenvironment is considered a key mechanism driving cancer progression and immune evasion (45, 46). Fujii Y et al. demonstrated that the cathepsin family plays a crucial role in modulating tumor immune cell dynamics—including immune cell recruitment, antigen processing and presentation, differentiation, and apoptosis—thereby contributing to the remodeling of the TME and facilitating immune suppression and tumor immune escape (47). Consistent with these findings, our study demonstrated that high CTSZ expression is significantly associated with infiltration of immunosuppressive cells, particularly M2-polarized macrophages and regulatory T cells (Tregs). Previous studies have confirmed that M2 macrophages inhibit antitumor immune responses through the secretion of immunosuppressive cytokines such as IL-10 and TGF-β (48), while Tregs support tumor immune evasion by suppressing dendritic cell maturation and effector T cell activity (49). We therefore hypothesize that CTSZ may contribute to the formation of an immunosuppressive TME by enhancing the recruitment and activation of these immune-inhibitory cell types, potentially through modulation of tumor-derived chemokines or downstream signaling pathways. This immunomodulatory function of CTSZ may in turn facilitate malignant progression in PCa.

Notably, our study revealed a significant upregulation of immune checkpoint molecules PD-1 and PD-L1 in PCa tissues with high CTSZ expression, suggesting that a subset of patients may exist in a distinct immunological state characterized by concurrent immune activation and exhaustion. This phenomenon has also been observed in other tumor types, such as clear cell renal cell carcinoma and hepatocellular carcinoma, where a co-occurrence of M2-polarized macrophages, regulatory T cells (Tregs), and elevated PD-1/PD-L1 expression has been reported. This immunosuppressive microenvironment is thought to contribute significantly to the limited responsiveness to immune checkpoint inhibitor therapies in these malignancies (50, 51).These findings imply that for PCa patients with high CTSZ expression and this immunosuppressive phenotype, combination therapeutic strategies—such as simultaneously targeting tumor-associated macrophages (TAMs), Tregs, and the PD-1/PD-L1 axis—may be required to enhance immunotherapeutic efficacy. Wu et al. have emphasized the growing importance of theragnostics, a strategy integrating diagnosis and therapy, in advancing precision medicine across major disease areas (52). Given the strong association of high CTSZ expression with both enhanced tumor aggressiveness and an immunosuppressive TME in PCa, CTSZ may serve not only as a prognostic biomarker but also as a therapeutic target within a theragnostic framework. This dual functionality positions CTSZ as a potentially valuable molecule for individualized treatment planning in PCa.

Beyond its immunomodulatory effects, our study also uncovered a strong correlation between elevated CTSZ expression and increased TMB, particularly *TP53* mutations, which are well known to contribute to genomic instability and tumor progression. Prior research has established the association of *TP53* mutations with therapeutic resistance, metastasis, and poor clinical outcomes across multiple cancer types (53). Our data further suggest that CTSZ may facilitate tumor progression in PCa by activating key oncogenic pathways, including STAT, VEGF, and CAM signaling. Future functional studies targeting these pathways could provide mechanistic insights into how CTSZ promotes malignant transformation in genomically unstable prostate tumors.

This study has several limitations. First, although our findings are supported by multiple independent cohorts and experimental validations, mechanistic insights remain limited. Specifically, the molecular pathway by which CTSZ regulates immune checkpoint molecules such as PD-1/PD-L1 requires further elucidation. Second, our *in vitro* assays may not fully recapitulate the complex TME observed *in vivo*. Third, although we propose CTSZ as a theragnostic target, clinical validation in patients receiving immunotherapy is necessary to confirm its predictive value. Future investigations should incorporate single-cell RNA sequencing (54), *in vivo* models, and functional rescue experiments to clarify upstream regulators and downstream mediators of CTSZ activity.

## 5 Conclusion

This study comprehensively demonstrates that CTSZ is significantly overexpressed in PCa and is closely associated with unfavorable clinicopathological features. Functional experiments confirmed its tumor-promoting roles in proliferation, migration, and invasion. Notably, CTSZ overexpression is linked to an immune-enriched yet immunosuppressive tumor microenvironment, characterized by increased infiltration of regulatory T cells and M2 macrophages, as well as upregulation of immune checkpoint molecules such as PD-1 and PD-L1. These findings suggest a potential role for CTSZ in immune evasion and checkpoint regulation. Additionally, high CTSZ expression correlates with increased tumor mutation burden and *TP53* mutations, further supporting its involvement in tumor progression. Gene enrichment analyses identified CAM, VEGF, and STAT pathways as potential mediators of its oncogenic function. Overall, CTSZ may serve as a robust prognostic biomarker and therapeutic target in PCa, with potential implications for guiding immunotherapy and precision medicine strategies.

## Data Availability

The datasets presented in this study can be found in online repositories. The names of the repository/repositories and accession number(s) can be found in the article/**Supplementary Material**.
