# Supplementary material for: Prognostic and immunological implications of cathepsin Z overexpression in prostate cancer
Source: Front Immunol. 2025 Jun 11;16:1618487. doi: 10.3389/fimmu.2025.1618487 (PMC12187850; doi:10.3389/fimmu.2025.1618487)
Supplement: Supplementary file 1 [file Table1.docx]

Supplementary Material

**Table S1. Detailed clinical features of AHMU-PC cohort**

| **Patient ID** | **Age** | **TPSA** | **survival status** | **Recurrence-free survival time, moths** | **Gleason score** | **Pathology T Stage** |
| --- | --- | --- | --- | --- | --- | --- |
| 1 | 62 | 12.23 | Non-recurrence | 37.02 | NA | T2 |
| 2 | 61 | 11.88 | Non-recurrence | 49.44 | 7 | T2 |
| 3 | 72 | 14.93 | Non-recurrence | 59.15 | 7 | T2 |
| 4 | 77 | 67.36 | Recurrence | 21.54 | 7 | T2 |
| 5 | 79 | 29.55 | Non-recurrence | 19.67 | 7 | T3 |
| 6 | 83 | 47.98 | Non-recurrence | 12.16 | 7 | T2 |
| 7 | 76 | 11.51 | Non-recurrence | 3.38 | 7 | T2 |
| 8 | 65 | 12.41 | Non-recurrence | 23.9 | 6 | T2 |
| 9 | 68 | 8.47 | Recurrence | 29.15 | 6 | T2 |
| 10 | 56 | 5.09 | Non-recurrence | 68.23 | 7 | T2 |
| 11 | 71 | 77.16 | Recurrence | 46 | 8 | T2 |
| 12 | 73 | 9.14 | Non-recurrence | 17.84 | 6 | T2 |
| 13 | 76 | 9.42 | Recurrence | 47.02 | 6 | T2 |
| 14 | 78 | 2 | Non-recurrence | 48.62 | 6 | T2 |
| 15 | 75 | 10.73 | Non-recurrence | 20.69 | 7 | T3 |
| 16 | 73 | 9.48 | Non-recurrence | 5.28 | 8 | T2 |
| 17 | 63 | 1.84 | Recurrence | 13.57 | 9 | T2 |
| 18 | 70 | 7.38 | Non-recurrence | 50.79 | 6 | T2 |
| 19 | 75 | 94.74 | Recurrence | 23.64 | 9 | T2 |
| 20 | 77 | 3.63 | Recurrence | 4.43 | 9 | T3 |
| 21 | 54 | 3.67 | Recurrence | 2.46 | 9 | T3 |
| 22 | 54 | 3.92 | Recurrence | 27.9 | 8 | T2 |
| 23 | 78 | 10.84 | Recurrence | 21.57 | 7 | T4 |
| 24 | 68 | 100 | Non-recurrence | 6.3 | 9 | T2 |
| 25 | 67 | 6.41 | Recurrence | 65.9 | 7 | T2 |
| 26 | 70 | 13.73 | Non-recurrence | 10.2 | 7 | T2 |
| 27 | 65 | 0.704 | Recurrence | 21.51 | 9 | T2 |
| 28 | 77 | 15.2 | Recurrence | 30.89 | 6 | T2 |
| 29 | 63 | 8.77 | Non-recurrence | 11.67 | 6 | T2 |
| 30 | 69 | 5.13 | Recurrence | 63.97 | 6 | T2 |
| 31 | 69 | >100 | Non-recurrence | 10.52 | 9 | T2 |
| 32 | 63 | NA | Recurrence | 6.85 | NA | T2 |
| 33 | 80 | 18.61 | Recurrence | 23.64 | 8 | T2 |
| 34 | 72 | 28.902 | Non-recurrence | 40.89 | 8 | T2 |
| 35 | 78 | 5.98 | Non-recurrence | 27.64 | 7 | T2 |
| 36 | 74 | 33.85 | Non-recurrence | 40.33 | 9 | T2 |
| 37 | 68 | 14.72 | Recurrence | 16.26 | 9 | T2 |
| 38 | 62 | 72.67 | Non-recurrence | 52.33 | 9 | T2 |
| 39 | 49 | 100 | Recurrence | 61.44 | 9 | T2 |
| 40 | 70 | 41.74 | Recurrence | 36.03 | 10 | T3 |
| 41 | 73 | 61.3 | Non-recurrence | 25.34 | 7 | T2 |
| 42 | 72 | 12.31 | Non-recurrence | 47.08 | 7 | T3 |
| 43 | 78 | 100 | Non-recurrence | 58.03 | 7 | T2 |
| 44 | 63 | 6.26 | Non-recurrence | 57.51 | 6 | T3 |
| 45 | 59 | 45.98 | Non-recurrence | 9.38 | 8 | T3 |
| 46 | 72 | 20.59 | Non-recurrence | 67.38 | 7 | T2 |
| 47 | 75 | 15.2 | Non-recurrence | 26.49 | 6 | T2 |
| 48 | 52 | 15.92 | Recurrence | 4.43 | 8 | T2 |
| 49 | 72 | 30.52 | Recurrence | 32.49 | NA | T3 |
| 50 | 73 | 8.33 | Recurrence | 33.41 | 8 | T2 |
| 51 | 85 | 95.59 | Non-recurrence | 43.8 | 8 | T2 |
| 52 | 71 | 14.93 | Non-recurrence | 10.36 | 9 | T3 |
| 53 | 63 | 8.38 | Non-recurrence | 18.03 | 8 | T2 |
| 54 | 70 | 100 | Recurrence | 40.49 | 10 | T2 |
| 55 | 76 | 6.34 | Non-recurrence | 79.02 | 6 | T2 |
| 56 | 82 | 42.67 | Recurrence | 56.85 | 8 | T2 |
| 57 | 53 | 8.07 | Non-recurrence | 25.41 | 7 | T2 |
| 58 | 78 | 10.56 | Recurrence | 58.52 | 6 | T2 |
| 59 | 66 | 11.63 | Non-recurrence | 30.85 | 7 | T2 |
| 60 | 67 | 6.01 | Non-recurrence | 28.39 | 6 | T2 |
| 61 | 75 | 13.34 | Recurrence | 12.16 | 7 | T2 |
| 62 | 72 | 23.11 | Recurrence | 54.46 | 9 | T2 |
| 63 | 66 | 9.99 | Non-recurrence | 67.67 | 7 | T2 |
| 64 | 56 | 100 | Non-recurrence | 7.05 | 8 | T3 |
| 65 | 78 | 10.56 | Recurrence | 58.52 | 6 | T2 |
| 66 | 54 | 88.12 | Recurrence | 35.08 | 9 | T4 |
| 67 | 77 | 6.68 | Recurrence | 28.03 | 7 | T3 |
| 68 | 62 | 9.26 | Non-recurrence | 1.61 | 8 | T2 |
| 69 | 52 | 12.96 | Recurrence | 30.56 | 6 | T2 |

**Table S2. Clinicopathological features of enrolled cohorts.**

|  | **TCGA-PRAD** | **MSKCC** | **AHMU-PC** |
| --- | --- | --- | --- |
| Patients’ number | 496 | 140 | 69 |
| Age, years old | 61.02 ± 6.86 | 58.13 ± 6.97 | 69.16 ± 8.43 |
| Gleason^*^ |  |  |  |
| 6 | 45 | 41 | 16 |
| 7 | 241 | 76 | 21 |
| 8 | 61 | 11 | 13 |
| 9 | 136 | 10 | 14 |
| 10 | 3 | - | 2 |
| PSA^#^, ng/dl |  |  |  |
| ≤ 10 | 415 | 114 | 29 |
| > 10 | 16 | 24 | 39 |
| Stage^†^ |  |  |  |
| T1 | - | - | - |
| T2 | 184 | 86 | 55 |
| T3 | 286 | 47 | 12 |
| T4 | 9 | 7 | 2 |

*In the TCGA-PRAD cohort, 10 cases lacked Gleason scores, 65 cases were missing PSA values, and 17 cases had no recorded T stage information. *In the MSKCC cohort, Gleason scores were unavailable for 2 cases, and PSA values were missing for another 2 cases. *In the AHMU-PC cohort, Gleason scores were missing for 3 cases, and PSA values were unavailable for 1 case.

**Table S3.** **Primer and siRNA sequences used in this study.**

| **No.** | **Primer Name** | **Type** | **Sequence(5'to3')** |
| --- | --- | --- | --- |
| 1 | siRNA-1 | Sense | GGAUCAACAUCAAGAGGAATT |
|  |  | Anti-Sense | UUCCUCUUGAUGUUGAUCCTT |
| 2 | siRNA-2 | Sense | GGAAUAAUGGCAACAGAAATT |
|  |  | Anti-Sense | UUUCUGUUGCCAUUAUUCCTT |
| 3 | siRNA-3 | Sense | GCAACAACUACCAGGCCAATT |
|  |  | Anti-Sense | UUGGCCUGGUAGUUGUUGCTT |
| 4 | CTSZ_F | Forward | CAGCGGATCTGCCCAAGAG |
| 5 | CTSZ_R | Reverse | CGATGACGTTCTGCACGGA |
| 6 | GAPDH_F | Forward | CAGGAGGCATTGCTGATGAT |
| 7 | GAPDH_R | Reverse | GAAGGCTGGGGCTCATTT |
